# Semi-continuous pilot-scale microbial oil production with *Metschnikowia pulcherrima* on starch hydrolysate

**DOI:** 10.1186/s13068-020-01756-2

**Published:** 2020-07-16

**Authors:** Felix Abeln, Robert H. Hicks, Hadiza Auta, Mauro Moreno-Beltrán, Luca Longanesi, Daniel A. Henk, Christopher J. Chuck

**Affiliations:** 1 Centre for Sustainable and Circular Technologies, University of Bath, Bath, UK; 2 Department of Chemical Engineering, University of Bath, Bath, UK; 3 Department of Biology & Biochemistry, University of Bath, Bath, UK

**Keywords:** Hydrolysed starch, Industrial biotechnology, *Metschnikowia pulcherrima*, Microbial lipid, Oleaginous yeast, Pilot scale, Scale-up

## Abstract

**Background:**

Heterotrophic microbial oils are potentially a more sustainable alternative to vegetable or fossil oils for food and fuel applications. However, as almost all work in the area is conducted on the laboratory scale, such studies carry limited industrial relevance and do not give a clear indication of what is required to produce an actual industrial process. *Metschnikowia pulcherrima* is a non-pathogenic industrially promising oleaginous yeast which exhibits numerous advantages for cost-effective lipid production, including a wide substrate uptake, antimicrobial activity and fermentation inhibitor tolerance. In this study, *M.* *pulcherrima* was fermented in stirred tank reactors of up to 350 L with 250-L working volume in both batch and semi-continuous operation to highlight the potential industrial relevance. Due to being food-grade, suitable for handling at scale and to demonstrate the oligosaccharide uptake capacity of *M.* *pulcherrima*, enzyme-hydrolysed starch in the form of glucose syrup was selected as fermentation feedstock.

**Results:**

In batch fermentations on the 2-L scale, a lipid concentration of 14.6 g L^−1^ and productivity of 0.11 g L^−1^ h^−1^ were achieved, which was confirmed at 50 L (15.8 g L^−1^; 0.10 g L^−1^ h^−1^). The maximum lipid production rate was 0.33 g L^−1^ h^−1^ (daily average), but the substrate uptake rate decreased with oligosaccharide chain length. To produce 1 kg of dry yeast biomass containing up to 43% (w/w) lipids, 5.2 kg of the glucose syrup was required, with a lipid yield of up to 0.21 g g^−1^ consumed saccharides. In semi-continuous operation, for the first time, an oleaginous yeast was cultured for over 2 months with a relatively stable lipid production rate (around 0.08 g L^−1^ h^−1^) and fatty acid profile (degree of fatty acid saturation around 27.6% w/w), and without contamination. On the 250-L scale, comparable results were observed, culminating in the generation of nearly 10 kg lipids with a lipid productivity of 0.10 g L^−1^ h^−1^.

**Conclusions:**

The results establish the importance of *M.* *pulcherrima* for industrial biotechnology and its suitability to commercially produce a food-grade oil. Further improvements in the productivity are required to make *M.* *pulcherrima* lipid production industrial reality, particularly when longer-chain saccharides are involved.

## Background

The benefits of microbial oils to supply the food and fuel oil market, such as its potential sustainability, have been highlighted many times [1–3]. Despite enormous potential, to date only a few oleaginous yeast have been cultured at the pilot scale and above [4–12], largely due to not finding a suitable host able to produce bulk oils close to market price or with a composition to qualify as high-value niche oils, as well as the absence of suitable equipment. Consequently, complex techno-economic studies are often based on results from laboratory-scale fermentations [13, 14], hampering the significance of those findings. Moreover, the majority of those pilot-scale experiments are run in batch or fed-batch operation, additionally limiting data for other promising operation modes such as continuous or semi-continuous operation [3, 15]. On route to commercialisation, it is key to show that a suitable oleaginous yeast is scalable to underline its industrial attractiveness [10].

It has been shown that *Metschnikowia pulcherrima* is a very promising oleaginous yeast for commercial lipid production, growing on a variety of low-cost substrates in non-sterile environments [8, 16]. The growth on certain oligosaccharides has been established [16, 17], making complex hydrolysates from waste streams, lignocellulosic biomass or starch attractive substrates. As being food-grade, hydrolysed starch in the form of glucose syrup (GS) is potentially a suitable feedstock for the production of a food-grade microbial oil, though the economic feasibility would depend heavily on the characteristics and price of the produced oil and by-products [13, 14]. A few other oleaginous yeasts including *Rhodotorula toruloides* [18, 19] have been cultured on starch hydrolysates, often derived from the cassava plant [18–20], but also on untreated starch [21] or starch wastewater [5]. Despite starch hydrolysates qualifying as a food-grade material, the aforementioned studies target the production of biodiesel. Considering *M. pulcherrima*’s antagonistic traits facilitating sterility [22, 23] and capability of producing an oil similar in composition to prominent vegetable oils including palm oil [3, 16], the development of a food-grade oil can be envisioned in addition to biodiesel.

Culturing oleaginous yeasts in flow operation can benefit the process performance such as through  increasing the productivity or supplying a consistent product stream [3, 15]. With *M.* *pulcherrima*, a nearly twofold increase of lipid production rates has been achieved in semi-continuous and continuous operation in combination with cell densities above 100 g L^−1^ [3]. However, changes in the cell morphology have been observed after around 10 days of cultivation [3, 24], which presumably must be overcome to provide a consistent product stream. To highlight the industrial relevance of the yeast, three aspects were addressed in this study:

- Assess the growth of *M. pulcherrima* on maltooligosaccharides.
- Achieve a consistent product stream in a semi-continuous culture (over 60 days).
- Demonstrate the scalability of *M. pulcherrima* (up to 250 L).

## Results and discussion

### Maltooligosaccharide uptake by *M. pulcherrima*

To assess the growth of *M. pulcherrima* on the maltooligosaccharides composing GS, batch fermentations were conducted on the 2- and 50-L scale, providing an insight into the scalability of *M. pulcherrima*. As nitric acid has been routinely used in previous stirred tank reactor fermentations with *M. pulcherrima* [3, 16, 25], but phosphoric acid being safer to handle at scale, the impact of both pH control agents on the fermentation parameters was additionally investigated.

**Fig. 1:**
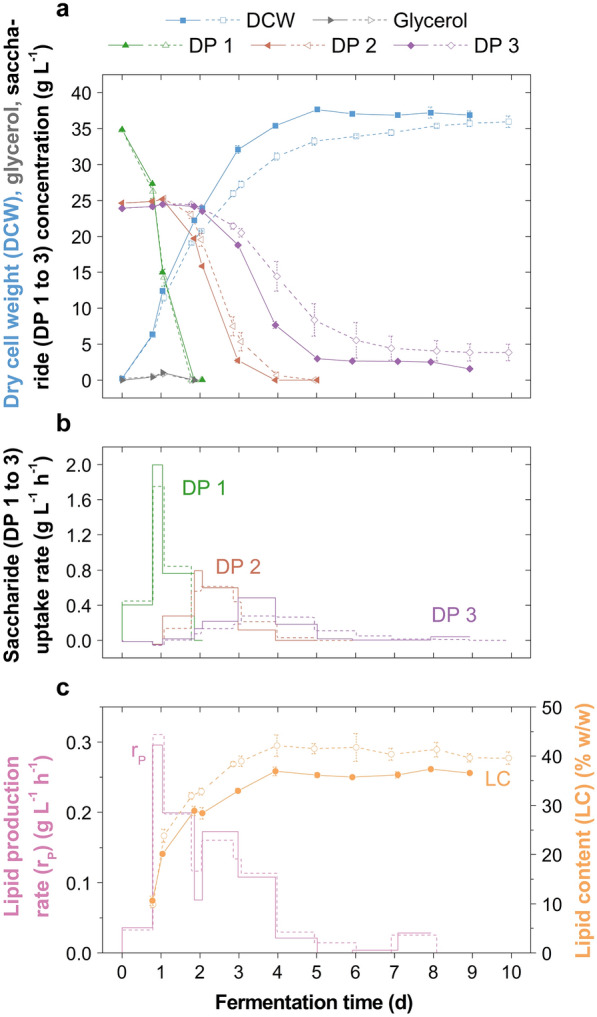
Batch cultivation of *Metschnikowia pulcherrima* on glucose syrup on the 2-L scale. **a** Profiles of dry cell weight, glycerol and saccharide concentrations, **b** saccharide uptake rates, and **c** lipid production rate and lipid content in stirred tank reactor fermentations of an evolved *M.* *pulcherrima* strain on glucose syrup and yeast extract (duplicate, mean ± standard error). Dashed lines and empty symbols: nitric acid pH regulation; solid lines and filled symbols: phosphoric acid pH regulation. For clarity, error bars are omitted where rates are displayed. *DP* degree of polymerisation

In batch fermentations on the 2-L scale, *M. pulcherrima* assimilated glucose (DP 1), maltose (DP 2) and maltotriose (DP 3), the latter two being broken down simultaneously after glucose depletion suggesting that a similar metabolic pathway is involved (Fig. 1). The amylolytic activity of *M. pulcherrima* isolates has been demonstrated previously [26]. The corresponding enzyme is unlikely an α-amylase, but instead the breakdown is potentially facilitated intracellularly by an α-glucosidase (maltase) such as with *Saccharomyces cerevisiae* [26, 27]. On the 24% (w/v) GS supplied, the yeast grew to a dry cell weight (DCW) of 37.8 g L^−1^ and produced lipids up to a concentration of 14.6 g L^−1^ and yield of 0.18 g g^−1^ consumed saccharides. The uptake of the produced glycerol (≤ 1.0 g L^−1^) was favoured over maltose assimilation, and no additional glycerol was detected upon metabolisation of DP 2 and DP 3 (Fig. 1a). Arabitol, another polyol produced by *M. pulcherrima* [3, 25], was produced in lower quantities (≤ 0.2 g L^−1^) and present throughout the fermentation.

Saccharide chain length had a distinct effect on the fermentation kinetics, with maximum saccharide uptake rates decreasing from 2.0 (DP 1) to 0.8 (DP 2) and 0.5 g L^−1^ h^−1^ (DP 3), consequently decreasing biomass production rates over time (Fig. 1). With amylolytic enzymes typically exhibiting increased kinetics with increasing maltooligosaccharide chain length [28], reaction rates are presumably limited by the transport efficiencies [27]. Consequently, the yeast achieved maximum biomass and lipid production rates of 0.98 and 0.31 g L^−1^ h^−1^ (average over 6.8 h), respectively, during the growth on glucose. These reaction rates are higher than those reported during batch growth on glucose in nitrogen-limited broth (0.72 and 0.25 g L^−1^ h^−1^, respectively [3]), but this was impacted by the higher frequency of sampling and process parameters influencing fermentation kinetics such as the higher amount of yeast extract supplied initially [25] and the use of phosphoric acid for pH control (Fig. 1).

Indeed, compared to using phosphoric acid, nitric acid pH control led to a higher lipid content (Fig. 1c). Whilst similar lipid concentrations were obtained with both acids, the use of phosphoric acid led to advanced fermentation kinetics (Fig. 1a) and was therefore used in subsequent fermentations in this study.

**Fig. 2:**
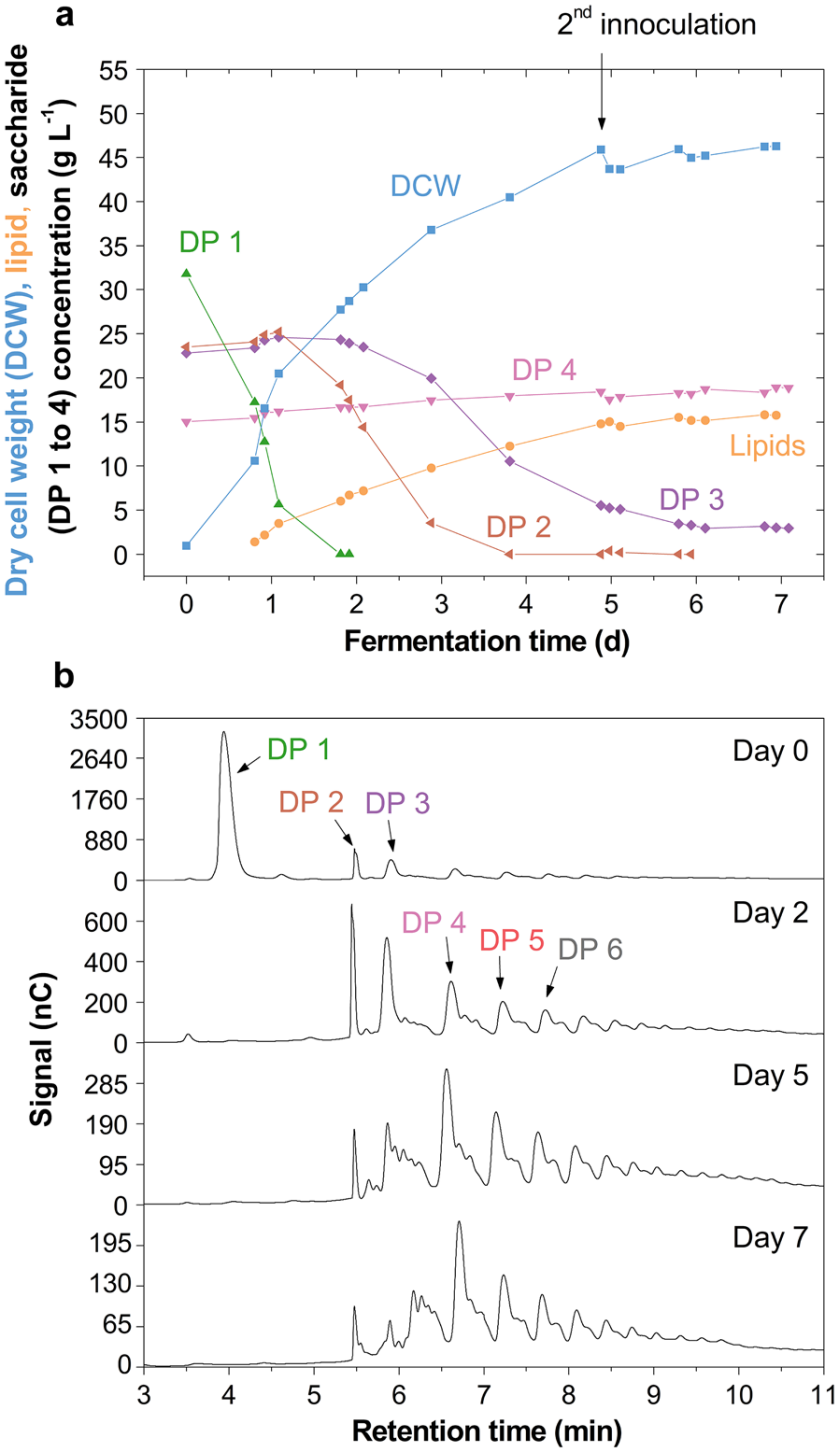
Batch cultivation of *Metschnikowia pulcherrima* on glucose syrup on the 50-L scale. **a** Profiles of dry cell weight, lipid and saccharide concentrations, and **b** signals obtained through high-performance anion-exchange chromatography of fermentation samples, in stirred tank reactor fermentation of an evolved *M.* *pulcherrima* strain on glucose syrup and yeast extract (singlicate). After 5 days, a second inoculum was added to the reactor to promote oligosaccharide uptake. *DP* degree of polymerisation

On the 50-L scale, an 18% (w/w) higher DCW was achieved compared to the 2-L scale, but the lower lipid content (34.3% w/w) meant only a slightly higher lipid concentration of 15.8 g L^−1^ was obtained (Fig. 2, Table 1). Such behaviour has also been observed in the scale-up with *Rhodotorula diobovata* from 7 to 150 L [6] and is potentially due to the differences in reactor design. Remarkably, the highest yet reported lipid yield of *M.* *pulcherrima* was achieved, amounting to 0.21 g g^−1^ consumed saccharides. This is considerably higher compared to growth on synthetic media including glucose (0.15 g g^−1^ [3]) or glycerol (0.10 g g^−1^ [8]) as carbon sources. Maximum lipid production (0.33 g L^−1^ h^−1^) and DP 1 to 3 saccharide uptake (1.8, 0.7 and 0.4 g L^−1^ h^−1^) rates were very similar to those at the 2-L scale. *M.* *pulcherrima* may also be able to break down the long-chain oligosaccharides (Fig. 2b). However, due to inefficient uptake of long-chain saccharides, the substrate utilisation was limited to 0.19 g g^−1^ (Table 1).

**Table 1:** Results from batch and semi-continuous cultivations of *M. pulcherrima* on glucose syrup

Operation specification	*V*_w_ (L)	*t*_f_ (d)	*X*_max_ (g L^−1^)	*L*_max_ (g L^−1^)	LC_max_ (% w/w)	*r*_S, max_ (g L^−1^ h^−1^)	*r*_X, max_ (g L^−1^ h^−1^)	*r*_L, max_ (g L^−1^ h^−1^)	*P*_X_ (g L^−1^ h^−1^)	*P*_L_ (g L^−1^ h^−1^)	*Y*_L_ (g g^−1^)	*U*_X/GS_ (g g^−1^)
Batch												
Nitric acid	2	9	36.0 ± 0.7	14.6 ± 0.6	42.6 ± 2.1	1.75 ± 0.03	0.76 ± 0.05	0.31 ± 0.03	0.16 ± 0.01	0.06 ± 0.00	0.18 ± 0.01	0.16 ± 0.00
Phosphoric acid	2	4	37.8 ± 0.2	14.0 ± 0.2	37.6 ± 0.2	2.00 ± 0.04	0.98 ± 0.05	0.30 ± 0.01	0.39 ± 0.00	0.11 ± 0.00	0.17 ± 0.00	0.15 ± 0.00
Phosphoric acid	50	7	46.3	15.8	34.3	1.78	1.22	0.33	0.28	0.10	0.21	0.19
Semi-continuous												
*D* = 0.21 d^−1^, no cells	2	22	43.4	14.0	33.0	1.46	0.75	0.25	0.33	0.10	0.15	0.10
*D* = 0.21 d^−1^, plus cells	2	22	43.2 ± 0.3	14.3 ± 0.1	33.7 ± 0.7	1.22 ± 0.04	0.81 ± 0.02	0.24 ± 0.02	0.35 ± 0.01	0.10 ± 0.01	0.15 ± 0.00	0.10 ± 0.00
*D* = 0.21 d^−1^, plus cells	250	16	40.0	11.6	32.6	1.65	0.74	0.30	0.33	0.10	0.16	0.10
*D* = 0.14 d^−1^, plus cells	2	62	50.8	16.0	34.1	1.21	0.75	0.18	0.26	0.08	0.14	0.12

In batch operation different pH control agents (nitric and phosphoric acid) and volumes (2 and 50 L) were compared, and in semi-continuous operation different dilution rates (*D*), cell addition patterns and volumes (2 and 250 L). For the cultivations at different scales, a constant gas volumetric flow rate was used as scale-up criterion and linear scale-up parameters kept constant as base for scale comparison [29]. The comparability of results from batch and semi-continuous fermentations is limited as different nutrient and inoculation conditions were applied (please see [3] for a detailed comparison). For duplicate experiments the mean value ± standard error is denoted

*V*_*w*_ working volume, *t*_*f*_ fermentation run time (for batch: fermentation time at* X*_max_; for semi-continuous: total fermentation time), *X* dried biomass concentration (as dry cell weight), *L* lipid concentration, *LC* lipid content, *r*_*S*_ saccharide uptake rate, *r*_*X*_ biomass production rate, *r*_*L*_ lipid production rate, *P*_*X*_ biomass productivity, *P*_*L*_ lipid productivity, *Y*_*L*_ lipid yield (per consumed saccharides), *U*_***X/GS***_ substrate utilisation (mass of dried biomass generated per mass of glucose syrup supplied)

Overall the excellent capability of *M. pulcherrima* to break down maltooligosaccharides, particularly up to DP 3, was demonstrated, achieving a remarkably high yield on the consumed saccharides. However, reaction rates decreased with chain length, and this imposes a trade-off between productivity and substrate utilisation. This is particularly important for a semi-continuous process, in which the dilution rate may be adjusted accordingly.

### Steady-state semi-continuous cultivation on the 2-L scale

To illustrate the productivity/yield trade-off, three semi-continuous fermentations were set up with a dilution rate (*D*) of 0.21 d^−1^ and one fermentation with *D* = 0.14 d^−1^. In two of the former (duplicate experiments) and the latter, additional preculture was added to the vessel together with the feed. Through this, it was attempted to mitigate the negative influence of the small cell formation typically observed in *M. pulcherrima* cultures after around 10 days [3, 24]. The overall goal was to achieve a steady output of lipids with a consistent composition.

**Fig. 3:**
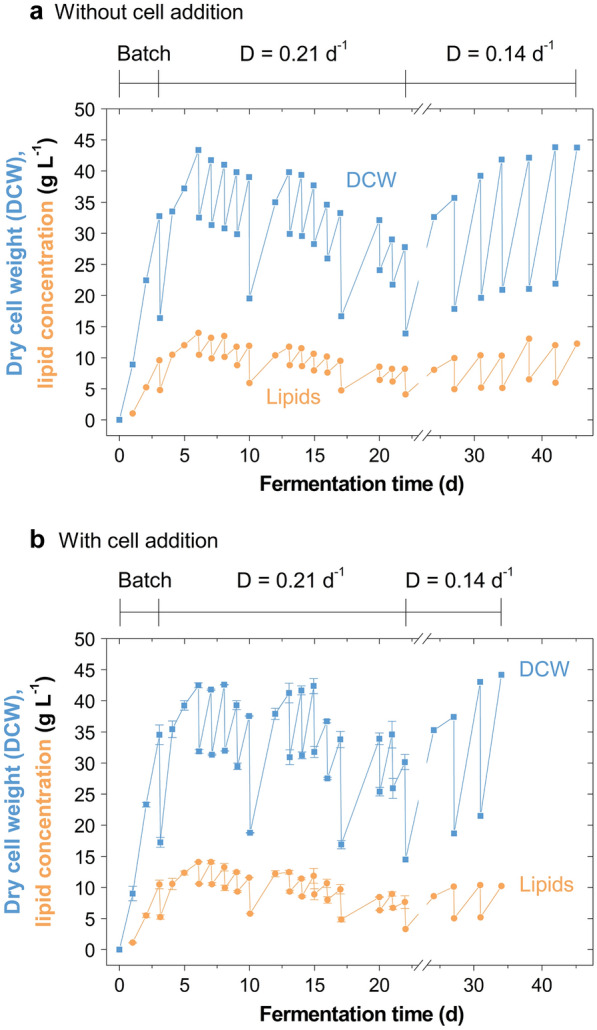
Semi-continuous cultivation of *Metschnikowia pulcherrima* on glucose syrup at a high dilution rate on the 2-L scale. **a** Profiles of dry cell weight and lipid concentration of an evolved *M.* *pulcherrima* strain cultured semi-continuously in stirred tank reactors at a dilution rate *D* = 0.21 d^−1^ on glucose syrup and yeast extract (singlicate), and **b** when additionally preculture was added with every feed (duplicate, mean ± standard error). After 22 days the dilution rate was switched to *D* = 0.14 d^−1^ (singlicate)

When fermenting at *D* = 0.21 d^−1^, the maximum biomass and lipid production rates were 0.81 and 0.25 g L^−1^ h^−1^ (daily average), respectively, an increase of up to 43% (w/w) from the initial batch (Fig. 3). The maximum biomass and lipid concentrations were 43.4 and 14.3 g L^−1^, respectively (Table 1). The small standard error between the duplicates demonstrates excellent repeatability for experiments with this yeast in stirred tank reactors, even in semi-continuous processing for over 3 weeks cultivation. Due to the high frequency of the feed, the yeast largely grew on glucose, with oligosaccharides accumulating in the broth (Additional file 1: Fig. S1). Therefore, compared to applying a lower dilution rate (*D* = 0.14 d^−1^), the biomass and lipid productivity were higher, but the substrate utilisation lower (Table 1).

The formation of small cells evident in the abruptly dropping average cell size occurred around Day 10 (Additional file 1: Figs. S2, S3). This was also observed in high-density flow cultures of *M. pulcherrima* on synthetic glucose medium [3]. Interestingly, the addition of fresh cells with every feed did not make a notable difference to this phenomenon. Possible reasons are the accumulation of certain nutrients or metabolites affecting the cell morphology of *M. pulcherrima* [3, 24]. This abrupt change in population dynamics did not majorly affect the DCW, but rather a general DCW decrease was observed (Fig. 3). Without the addition of preculture, this decrease was steadier, whereas with cell addition only notable towards the end of a 5-day consecutive broth exchange. Consequently, through the addition of preculture, the biomass productivity until Day 22 could be increased by 4.7 ± 1.2% (w/w). A similar decrease in biomass production and lipid content has been observed when culturing *Rhodotorula glutinis* semi-continuously on palm oil mill effluent [30]. Performing a broth exchange every 2 days, the lipid content distinctly dropped at a similar time as observed with *M. pulcherrima* (Day 9). Whilst the similarity between the behaviours is evident, morphological changes have not been reported in that study.

**Fig. 4:**
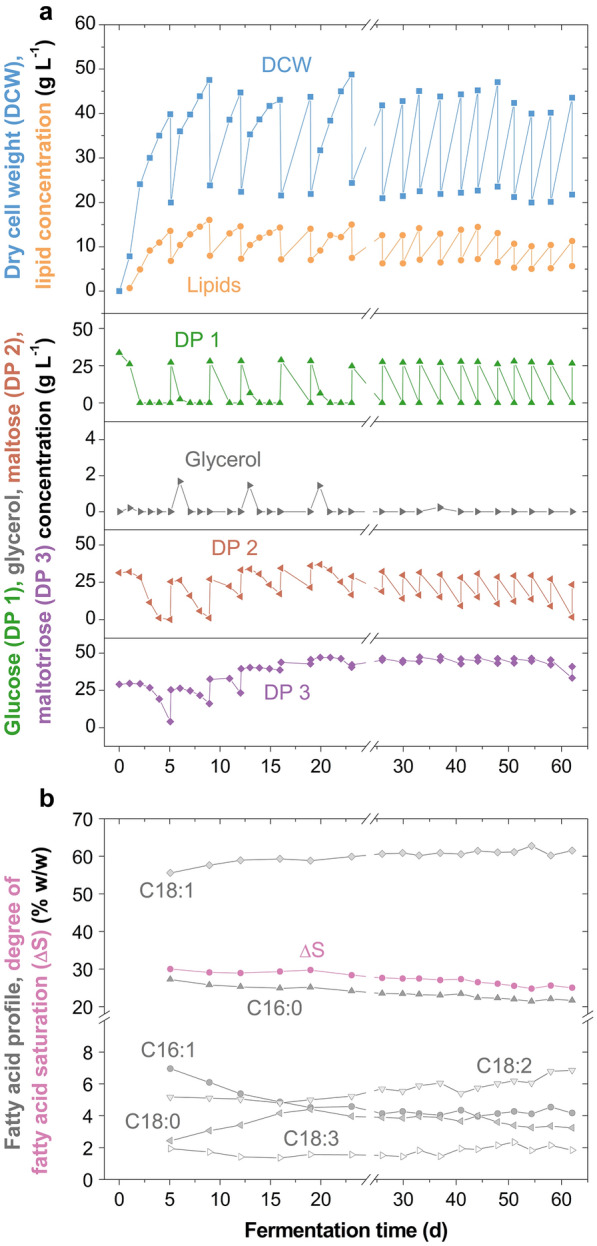
Semi-continuous cultivation of *Metschnikowia pulcherrima* on glucose syrup at a lower dilution rate on the 2-L scale. **a** Profiles of dry cell weight, lipid and saccharide concentrations, and **b** fatty acid profile and degree of fatty acid saturation in semi-continuous cultivations of an evolved *M.* *pulcherrima* strain in stirred tank reactors at a dilution rate *D* = 0.14 d^−1^ on glucose syrup and yeast extract (singlicate). Additional preculture was added with every feed

When fermenting at *D* = 0.14 d^−1^, the DCW, lipid content and fatty acid profiles remained considerably stable for 62 days (Fig. 4). However, the lipid content slightly dropped from approximately 33 to 26% (w/w) and the degree of fatty acid saturation from 30.0 to 25.1% (w/w). Through the lower feeding rate, a higher substrate uptake and hence, substrate utilisation was achieved compared to fermenting at *D* = 0.21 d^−1^ (Table 1). Indeed, the yeast grew on maltose and maltotriose (Fig. 4), but to achieve full conversion of these compounds an even lower dilution rate, for example, *D* = 0.07 d^−1^ (broth exchange every 7 days), would be required. Biomass and lipid productivities were held at around 0.26 and 0.08 g L^−1^ h^−1^, respectively, with the final lipid yield being 0.14 g g^−1^ consumed saccharides. Over time, the produced oil contained increasing C18:1 and C18:2 fatty acids at the expense of C16:0 (Fig. 4), which has been observed with *M.* *pulcherrima* previously [3], and is similar to other oleaginous yeasts [6]. Overall, through fermenting at a lower dilution rate the formation of small cells could not be avoided (Additional file 1: Fig. S4), but a drop in biomass productivity did not occur for over 2 months cultivation (Fig. 4). Remarkably, the lipid-rich cells grew to a diameter of up to 17.0 μm and the culture was not contaminated.

When reducing the dilution rate in fermentations from *D* = 0.21 to 0.14 d^−1^ (on Day 22), the previously dropping biomass production recovered (Fig. 3). This manifests that the higher dilution rate leads to the wash-out of this relatively slow-growing yeast. However, the instant recovery also means that is it possible to increase the productivity for short periods of time, by switching to a more frequent broth exchange, therefore reacting to market fluctuations or outages of other equipment, for instance (Table 1). A drop of the lipid content with increasing dilution rate has been observed with other oleaginous yeasts [30, 31].

Glycerol and arabitol have been proposed to serve as osmolytes with *M.* *pulcherrima* [3] and as such could have been expected in semi-continuous fermentations where saccharide concentrations > 300 g L^−1^ occurred (please see “Organism and media” and “Fermentation” sections). However, under the experimental conditions herein, these compounds were only detected extracellularly when the yeast grew on glucose (Fig. 4). Secreted glycerol was re-assimilated before maltose assimilation and thereafter only detected in small quantities (≤ 1.7 g L^−1^). It may be that the glycerol metabolism is carbon-source specific but more likely that any leached glycerol is re-assimilated by the yeast before it can be detected in the broth. Moreover, due to the saccharides split across more than six compounds and the higher molecular weights of the oligosaccharides, the water activity was always ≥ 0.98.

### Lipids from semi-continuous cultivation at the 250-L scale

**Fig. 5:**
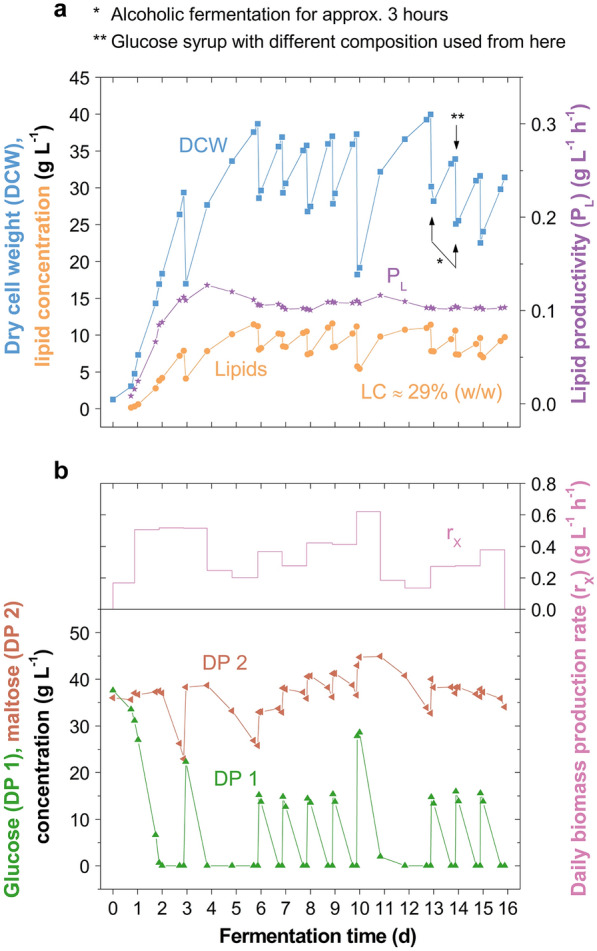
Semi-continuous cultivation of *Metschnikowia pulcherrima* on glucose syrup on the 250-L scale. **a** Profiles of dry cell weight, lipid concentration and lipid productivity (up to the corresponding time), and **b** profiles of glucose and maltose concentration, and the daily biomass production rate in semi-continuous stirred tank reactor fermentation at a dilution rate *D* = 0.21 d^−1^ of an evolved *M.* *pulcherrima* strain on glucose syrup and yeast extract (singlicate). Additional preculture was added with every feed

On the 250-L scale, the excellent scalability of *M. pulcherrima* as  determined on the 50-L scale (Table 1), was to be further demonstrated in semi-continuous culture. The goal was to achieve high productivities during a relatively short production time (16 days), wherefore a dilution rate of 0.21 d^−1^ was chosen. Consequently, the yeast mainly grew on glucose, with larger saccharides accumulating in the broth (Fig. 5). Maximum DCW, lipid content and concentration were 40.0 g L^−1^, 32.6% (w/w) and 11.6 g L^−1^, respectively. The lipid content remained stable throughout the cultivation with an average of 29.0% (w/w) from Day 3. On Day 10, at a lipid content of 28.6% (w/w), the dried yeast also contained 9.8% (w/w) crude protein, of which 81.2% were amino acids (Additional file 1: Table S1). Of these, 7.3% were lysine and 1.6% methionine, amino acids typically required in increased quantities in animal feed [32]. Whilst specifically the high lysine content is promising for use in animal feed, for instance as a soy protein substitute [33], the pepsin digestibility was low (50.6%).

### Scale comparison and productivity

In this study, *M. pulcherrima* was cultured in batch and semi-continuous operation on the 2-, 50- and 250-L scale (Table 1). Pilot-scale fermentations are often conducted to gather information on the scalability of an organism, such as in this study, and to determine scale-up criteria for further scale-up [10]. Exemplary scale-up criteria are constant power input, oxygen transfer coefficient or geometric similarity [29], though often the vessels are not designed to meet several criteria [34]. In this study, a constant gas volumetric flow rate was used as single scale-up criterion and linear scale-up parameters kept constant as base for scale comparison [29].

**Fig. 6:**
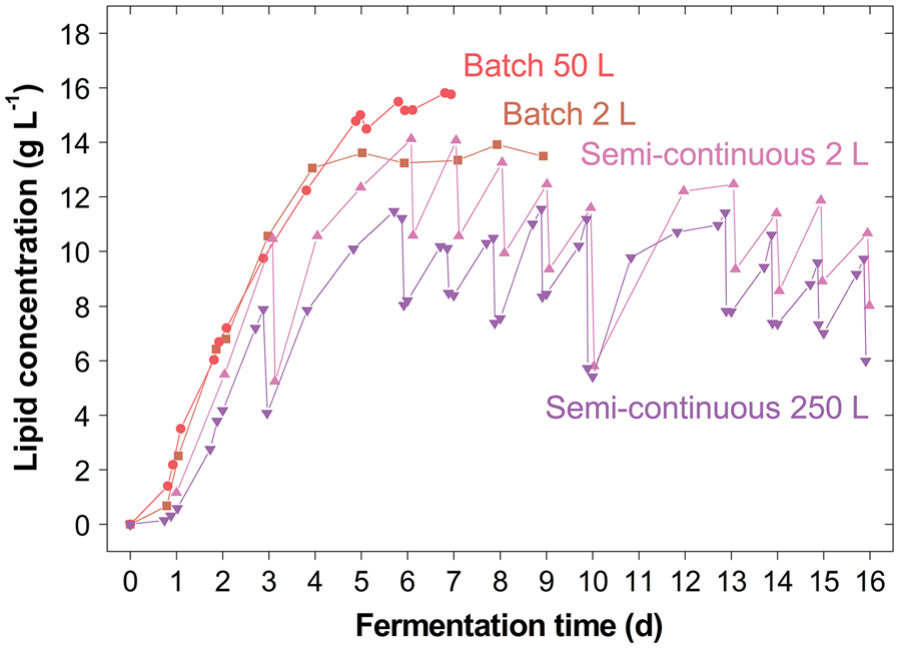
The scalability of *Metschnikowia pulcherrima*. Comparison of the lipid concentration in *M.* *pulcherrima* cultures when grown batch-wise or semi-continuously on glucose syrup on the 2-, 50- or 250-L scale in stirred tank reactors. A constant gas volumetric flow rate was used as scale-up criterion and linear scale-up parameters kept constant for each batch and semi-continuous operation as base for scale comparison [29]

Remarkably, throughout the different scales reported *M. pulcherrima* performed similarly well with respect to kinetic and yield parameters (Table 1). Although the lipid content was minorly compromised on the pilot scale, higher maximum lipid production rates were achieved. This remarkable scalability becomes even more apparent in the similar lipid concentration profiles (Fig. 6). Potentially, the small differences in the results could be further reduced when increasing the geometric similarity between the reactor systems [10, 29].

Despite the promising results at the 250-L scale, only a total of 9.8 kg oil was produced in semi-continuous cultivation, equivalent to a lipid productivity of 0.10 g L^−1^ h^−1^ (Fig. 5). It is generally recognised that for commercial lipid production productivities above 1 g L^−1^ h^−1^ are likely required [13, 14]. Therefore, whilst it was demonstrated that *M. pulcherrima* is a robust, and importantly, scalable organism, its key issue remains the low lipid productivity, particularly when longer chain saccharides are involved. A combination of strategies is therefore required to achieve lipid productivities suitable for commercial production [13]. This includes fermentation at high cell densities, through which *M.* *pulcherrima* has already been shown to achieve lipid productivities nearly double of those herein (0.18 g L^−1^ h^−1^) [3]. Through increased micronutrient supplementation, the lipid productivity in batch fermentation could be further increased to 0.29 g L^−1^ h^−1^ [25]. And finally, genetic modification is required for this very promising oleaginous yeast to attract further industrial relevance. An example is set with *Yarrowia lipolytica*, with which a lipid productivity of 0.92 g L^−1^ h^−1^, an approx. 11.5-fold increase from the wild type has been achieved in fed-batch operation after substantial genetic engineering [35].

## Conclusions

Hydrolysed starch was shown to be a suitable feedstock for *M.* *pulcherrima*, with the lipid yields with respect to the consumed saccharides considerably exceeding those using any other feedstock to date, including glucose and glycerol. To decrease process cost, the suitability of starchy wastes such as cassava pulp or starch wastewater could be investigated, though they might be unsuitable for producing a food-grade oil. Additionally, the oligosaccharide breakdown capacity and rates require improvement. The results demonstrate that *M. pulcherrima* is an oleaginous yeast suitable for continued operation, with a reasonably steady lipid production rate, fatty acid composition of the produced oil and no contamination in fermentation over 2 months. Excellent repeatability in lipid production parameters has been demonstrated in semi-continuous operation over 3 weeks. Finally, *M. pulcherrima* has proven itself as scalable oleaginous yeast, with superior biomass and lipid concentrations as well as lipid production rates achieved at the 50- and 250-L scale compared to the 2-L scale. The kinetics of this promising yeast require improvement, but through a combination of high-density cultivation, media supplementation and potentially genetic engineering and/or directed evolution, it is envisaged that lipid productivities required for commercial production can be achieved. These exciting results are valuable for techno-economic analysis of microbial lipid production at this scale and provide further credibility to the emergence of *M. pulcherrima* as industrially relevant yeast.

## Materials and methods

### Organism and media

Chemicals were purchased from Sigma-Aldrich unless noted otherwise. All fermentation equipment were sterilised and media autoclaved at 121 °C for 20 min prior to use. For the fermentation experiments, *M.* *pulcherrima* strain NCYC 4331 (National Collection of Yeast Cultures, Norfolk, UK), which has been evolved towards increased fermentation inhibitor tolerance [3], was used. The strain was kept as 20% (v/v) glycerol stock at − 80 °C. Precultures were prepared in 0.1- to 5-L Erlenmeyer (shake) flasks with 20% (v/v) working volume using soy–malt broth (SMB: soy peptone 30 g L^−1^; malt extract 25 g L^−1^; in deionised water; pH 5 with 6 M HCl), inoculated with 0.15% (v/v) defrosted *M.* *pulcherrima* glycerol stock and incubated at 25 °C and 180 rpm (Innova 4300, New Brunswick Scientific) for 24 h. The primary carbon source was food-grade (confectioner’s) glucose syrup (GS), obtained from the enzymatic hydrolysis of starch (HH Industries Ltd, UK). It was selected as carbon source since it is food-grade, suitable for handling at scale and contains a range of oligosaccharides; mimicking other complex feedstock such as lignocellulosic hydrolysates. The average composition of the GS with a dextrose equivalent of around 39.8 is given in Table 2. The macro- and micronutrients were supplied through yeast extract (YE). In this respect, it has been shown that the lipid content and productivity of *M. pulcherrima* can be increased using minimal medium with a high yeast extract content [8, 25].

**Table 2:** Characteristics of the glucose syrup used in this investigation

Specification	Saccharide	Value
DP 1	Glucose	147.3 ± 8.6 g g^−1^
DP 2	Maltose	121.1 ± 17.8 g g^−1^
DP 3	Maltotriose	114.8 ± 14.9 g g^−1^
DP 3 to 4	Unknown	66.0 ± 12.5 g g^−1^
DP 4 and 5	Maltotetraose and maltopentaose	85.6 ± 19.4 g g^−1^
≥ DP 6	Maltohexaose, maltoheptaose, etc.	332.0 ± 37.8 g g^−1^
DE	–	39.8 ± 0.7
Density (20 °C)	–	1.40 ± 0.02 g mL^−1^

Values are given as mean ± standard deviation

*DP* degree of polymerisation, *DE* dextrose equivalent

In this study, the batch and semi-continuous cultivations were conducted using different nutrient and inoculation conditions due to limitations in equipment available at scale. However, both operation modes have been compared in detail elsewhere [3]. The batch fermentation medium consisted of 24% (w/v) GS and 3 g L^−1^ YE and was inoculated with 4% (v/v) preculture. The initial batch fermentation medium for semi-continuous cultures was prepared as 24% (w/v) GS and 4.5 g L^−1^ YE and inoculated with 0.8% (v/v) preculture. The feed medium consisted of 40% (w/v) GS and 5 g L^−1^ YE. At the 2-L scale, deionised water was used for media preparation and at the 50- and 250-L scale, tap water. The pH of the media was adjusted to pH 4 as indicated in the following “Fermentation” section.

### Fermentation

Fermentation experiments were conducted in stirred tank reactors with a total volume of 2 L (2 L working volume; 2× Rushton impeller, micro-sparger 40 μm; Electrolab), 70 L (50 L; 2× Rushton impeller, ring sparger; Applikon) or 350 L (250 L; 2× Rushton impeller, ring sparger; Bioprocess Technology). The latter two are located at the BEACON Biorefining Centre of Excellence in Aberystwyth (UK). As base for scale comparison, the linear scale-up parameters temperature, pH, dissolved oxygen (DO), nutrient and inoculation conditions as well as the dilution rate (if applicable) were kept constant for each batch (2 and 50 L working volume) and semi-continuous (2 and 250 L) operation [29], and the same pH control agents (NaOH, H_3_PO_4_) were used throughout the scales. A constant gas volumetric flow rate (vvm) was used as single scale-up criterion [29] and the agitation rate was set to provide a constant DO. To this end, the fermentations were controlled at 20 °C, pH 4 (2 M NaOH, 1 M H_3_PO_4_ or HNO_3_) and DO 50% (0.5 vvm, 100–500 rpm)—conditions which have been shown suitable for maximum lipid yield and productivity [25]. Nitric acid was only used for pH control in 2-L batch fermentations to establish the suitability of using phosphoric acid for pH control, as in previous studies with *M.* *pulcherrima* nitric acid has routinely been used [3, 16, 25].

Batch fermentations were run for 7 days. After 5 days, another 4% (v/v) preculture was added to promote further oligosaccharide uptake. Sampling on the 2-L scale (around 5 mL) took place twice/day until glucose consumption, thereafter once/day, and on the 50-L scale (around 20 mL) three times/day (except on weekends once/day). Semi-continuous fermentations were initially started as a batch. The dilution rate *D* (in d^−1^) was calculated as:

$$D = V_{\text{w}} /\bar{\dot{V}}.$$

In this equation $V_{\text{w}}$ is the working volume (in L), and $\bar{\dot{V}}$ the average broth exchange per day (in L d^−1^). Three feeding regimes with different dilution rates and preculture addition patterns were used on the 2-L scale. The dilution rates and exchange volumes were chosen based on previous semi-continuous fermentations with *M. pulcherrima* [3] and its maximum substrate uptake rates under different nutrient conditions [25] as well on the glucose syrup feedstock (Table 1). The higher dilution rates (0.21 d^−1^) were applied to increase the productivity and the lower dilution rates (0.14 d^−1^) to increase the substrate utilisation.

1. *D* = 0.21 d^−1^ and additional preculture: On Day 3, 10 and 17, the broth was removed until 51.2% (v/v) of the working volume and 48% (v/v) feed medium as well as 0.8% (v/v) preculture were added. On Day 6, 13 and 20 as well as their three subsequent days, the broth was removed until 75.6% (v/v) and 24% (v/v) feed medium as well as 0.4% (v/v) preculture were added.
2. *D* = 0.21 d^−1^ and no additional preculture: On Day 3, 10 and 17, the broth was removed until 52% (v/v) of the working volume and 48% (v/v) feed medium added. On Day 6, 13 and 20, as well as their three subsequent days, the broth was removed until 76% (v/v) and 24% (v/v) feed medium added.
3. *D* = 0.14 d^−1^ and additional preculture: On Day 5, the broth was removed until 51.2% (v/v) of the working volume and 48% (v/v) feed medium as well as 0.8% (v/v) preculture were added. This was continued alternately every 4 or 3 days (i.e. Day 9, 12, 16, etc.) up to a total run time of 62 days.

After 22 days, feeding regimes 1 and 2 were switched to feeding regime 3 (*D* = 0.14 d^−1^) up to a total run time of 34 and 45 days, respectively. In the case of regime 2, no additional preculture was added (i.e. broth removal until 52% v/v instead of 51.2% v/v). On the 250-L scale, feeding regime 1 was used with 16 days run time. Sampling on the 2-L scale (around 5 mL) took place once/day and on the 250-L scale (around 20 mL) four times/day (except once/day on weekends). The removed broth was spun down with two parallel CEPA Z41 centrifuges at 2 L min^−1^ inlet flux, the biomass lyophilised and its composition analysed.

*Metschnikowia pulcherrima* is antagonistic to bacterial and fungal growth [22, 23], but possible bacterial contamination was visually assessed on micrograms and fungal/yeast contamination through plating approx. 10 μL culture out on iron-supplemented malt extract agar plates (MEA: agar 15 g L^−1^; malt extract 30 g L^−1^; mycological peptone 5 g L^−1^; plus 0.02 mg L^−1^ FeCl_3_) and evaluating the redness of the colonies after 3 days incubation at 25 °C [22]. *Metschnikowia pulcherrima* colonies can be differentiated through producing the red pigment pulcherrimin [22, 23] (Additional file 1: Fig. S5).

### Analytical methods

Yeast growth was assessed through the optical density (OD_600_) and DCW of the fermentation broth, the latter determined via centrifugation and overnight lyophilisation of the pellet from a known broth volume [16]. Cell size analysis was conducted according to reported procedures [24]. Briefly, images of cell culture, diluted to yield approximately 100 cells/image, were taken with an EVOS XL Cell Imaging System. The image was then processed with GIMP and Image J, where also the cell area was determined. This process was repeated until more than 300 cells were analysed. The lipid content of the dried biomass was determined with an adapted Bligh and Dyer [36] method, in which 40 to 80 mg dried cells were disrupted in 10 mL 6 M HCl at 80 °C for 1 h and the lipids extracted with an equal volume of chloroform/methanol (1:1 v/v) [16]. The fatty acid composition of the extracted lipids was ascertained through transesterification in methanol–sulphuric acid (1% v/v) at 90 °C for 2 h, extraction of the fatty acid methyl esters with hexane and subsequent gas chromatography [16]. The analysis of dried cell composition including protein analysis was performed by AB Agri (UK) according to standard procedures [33]. Briefly, moisture was determined through drying at 105 °C to constant weight, the crude protein (N × 6.25) by the Dumas [37] method, ash through incineration in a muffle furnace at 580 °C for 8 h, crude fibre through a fibre analyser (ANKOM 220, ANKOM), ether extract through Soxhlet extraction with diethyl ether, amino acids (AAs) through an amino acid analyser (AAA 500, INGOS), and the pepsin digestibility by the AOAC Method 971.09 using 0.02% pepsin.

The saccharides and metabolites in the fermentation broth were quantified via high-performance liquid chromatography (HPLC) using an ion-exclusion column (RHM-Monosaccharide H + (8%), Phenomenex) [3, 16]. Oligosaccharides with a degree of polymerisation (DP) of 3 to 7 were purchased for HPLC calibration from Dextra Laboratories (UK). Those with DP ≥ 7 had the same retention time, wherefore their concentration was estimated with the DP 7 standard. The dextrose equivalent (DE) was calculated as:

$${\text{DE }} = 100 \times \mathop \sum \limits_{n} \left( {x_{n} \times 180/\left( {180 \times n - 18 \times \left( {n - 1} \right)} \right)} \right).$$

In this equation, $x_{n}$ is the mass fraction of the saccharide with a DP of *n*. Qualitatively, the composition of saccharides was assessed using high-performance anion exchange chromatography with pulsed amperometric detection, employing a 250 mm × 4 mm CarboPac PA-100 column (Dionex) with the detailed conditions described elsewhere [38]. The water activities of the broth were calculated using Van’t Hoff, Raoult–Lewis and Ross equations as derived previously [3], taking into account the solubilised saccharides [39]. Concentrations in the broth were not rectified according to water evaporation or sampling.

The saccharide uptake rate *r*_S_ (in g L^−1^ h^−1^) was calculated as:

$$r_{\text{S}} = \left( {S_{1} - S_{2} } \right)/\left( {t_{1} - t_{2} } \right).$$

In this equation, *S*_1_ and *S*_2_ are the saccharide concentrations (in g L^−1^) at the consecutive sampling times *t*_1_ and *t*_2_ (in h), respectively. The biomass production rate *r*_X_ (in g L^−1^ h^−1^) was calculated as:

$$r_{\text{X}} = \left( {X_{2} - X_{1} } \right)/\left( {t_{2} - t_{1} } \right).$$

Here, *X*_1_ and *X*_2_ are the DCW (in g L^−1^) at the consecutive sampling times *t*_1_ and *t*_2_ (in h), respectively. The lipid production rate *r*_L_ (in g L^−1^ h^−1^) was calculated accordingly, with *X* substituted by the corresponding lipid concentration L (in g L^−1^). The biomass productivity *P*_X_ (in g L^−1^ h^−1^) was calculated as:

$$P_{\text{X}} = X_{\text{t}} /t_{\text{f}} .$$

In this equation, *X*_t_ is the total DCW produced (in g L^−1^) until the fermentation run time *t*_f_ (in h). For batch processes, t_f_ is the fermentation time at maximum DCW, and for semi-continuous processes, the total fermentation time, unless indicated otherwise. The lipid productivity *P*_L_ (in g L^−1^ h^−1^) was calculated accordingly, with *X*_t_ substituted by the total lipids produced *L*_t_ (in g L^−1^). The lipid yield *Y*_L_ (in g g^−1^) was calculated as:

$$Y_{\text{L}} = L_{\text{t}} /S_{\text{t}} .$$

Here, *S*_t_ is the total consumed saccharide concentration (in g L^−1^). The substrate utilisation *U*_X/GS_ (in g g^−1^) was calculated as:

$$U_{{{\text{X}}/{\text{GS}}}} = X_{\text{t}} /{\text{GS}}_{\text{t}} .$$

In this equation, GS_t_ is the total amount of glucose syrup supplied (in g L^−1^).

### Replication

Batch fermentations were conducted as duplicates on the 2-L and singlicate on the 50-L scale. In semi-continuous operation, the excellent repeatability of *M. pulcherrima* fermentation was further demonstrated through a 22-day duplicate fermentation (2-L scale, feeding regime 1), wherefore remaining 2- and 250-L semi-continuous fermentations were performed as singlicates. Errors are reported as the standard deviation in characterisation and standard error in biological experiments. For all duplicate fermentations, the standard error divided by the mean was less than 8% across all parameters depicted in Table 1.

## Supplementary information

**Additional file 1.** Additional table and figures.

## Data Availability

The datasets supporting the conclusions of this article are included within the article and the additional files.

## Abbreviations

*D*: Dilution rate (d^−1^)
DCW: Dry cell weight (g L^−1^)
DE: Dextrose equivalent (-)
DO: Dissolved oxygen (%)
DP: Degree of polymerisation
GS: Glucose syrup
GS_t_: Total amount of glucose syrup supplied (g L^−1^)
L: Lipid concentration (g L^−1^)
LC: Lipid content (% w/w)
*P*_L_: Lipid productivity (g L^−1^ h^−1^)
*P*_X_: Biomass productivity (g L^−1^ h^−1^)
*r*_L_: Lipid production rate (g L^−1^ h^−1^)
*r*_S_: Saccharide uptake rate (g L^−1^ h^−1^)
*r*_X_: Biomass production rate (g L^−1^ h^−1^)
*S*: Saccharide concentration (g L^−1^)
*S*_t_: Total consumed saccharide concentration (g L^−1^)
*t*_f_: Fermentation run time (h)
*U*_**X/GS**_: Substrate utilisation (g g^−1^)
$\bar{\dot{V}}$: Average broth exchange per day (L d^−1^)
*V*_w_: Working volume (L)
*X*: Dried biomass concentration (g L^−1^)
$x_{n}$: Mass fraction of the saccharide with a DP of *n* (g g^−1^)
YE: Yeast extract
*Y*_L_: Lipid yield (g g^−1^)
