# Supplementary material for: Semi-continuous pilot-scale microbial oil production with Metschnikowia pulcherrima on starch hydrolysate
Source: Biotechnol Biofuels. 2020 Jul 16;13:127. doi: 10.1186/s13068-020-01756-2 (PMC7367368; doi:10.1186/s13068-020-01756-2)
Supplement: Supplementary file 1 — Additional file 1. Additional table and figures. [file 13068_2020_1756_MOESM1_ESM.docx]

# Title page

**Additional information for**

**Semi-continuous pilot-scale microbial oil production with *Metschnikowia pulcherrima* on starch hydrolysate**

Felix Abeln,^†, ‡^ Robert H. Hicks,^§^ Hadiza Auta,^‡^ Mauro Moreno‐Beltrán,^§^ Luca Longanesi,^‡^ Daniel A. Henk,^§^ Christopher J. Chuck^‡, *^

† Centre for Sustainable and Circular Technologies, University of Bath, Bath, UK

‡ Department of Chemical Engineering, University of Bath, Bath, UK

§ Department of Biology & Biochemistry, University of Bath, Bath, UK

^*^ Corresponding author.

*E-mail address*: C.Chuck@bath.ac.uk (C. J. Chuck).

**This file includes on 7 pages (see navigation pane):**

Table S1

Figures S1 to S5

# Table S1

**Table S1:** Composition of *Metschnikowia pulcherrima* obtained from semi-continuous cultivation on glucose syrup on the 250 L scale (Day 10, Fig. 5). Focus is on the amino acid (AA) composition.

| Specification | Value (%) |
| --- | --- |
| Moisture | 2.6 |
| Crude protein (N x 6.25, Dumas) | 9.8 |
| Ash | 1.7 |
| Crude fibre | 0.6 |
| Oil A (ether extract) | 4.4 |
| Total oil (oil B) | 28.61 |
| Alanine | 0.53 |
| Arginine | 0.48 |
| Aspartic acid | 0.83 |
| Cystine | 0.14 |
| Glutamic acid | 1.17 |
| Glycine | 0.40 |
| Histidine | 0.20 |
| Iso-leucine | 0.39 |
| Leucine | 0.52 |
| Lysine | 0.58 |
| Methionine | 0.13 |
| Phenylalanine | 0.36 |
| Proline | 0.32 |
| Serine | 0.52 |
| Threonine | 0.62 |
| Tyrosine | 0.25 |
| Valine | 0.52 |
| Sum of AA | 7.96 |
| Sum of AA/protein | 81.20 |
| Pepsin digestibility (0.02%) | 50.6 |

# Figure S1


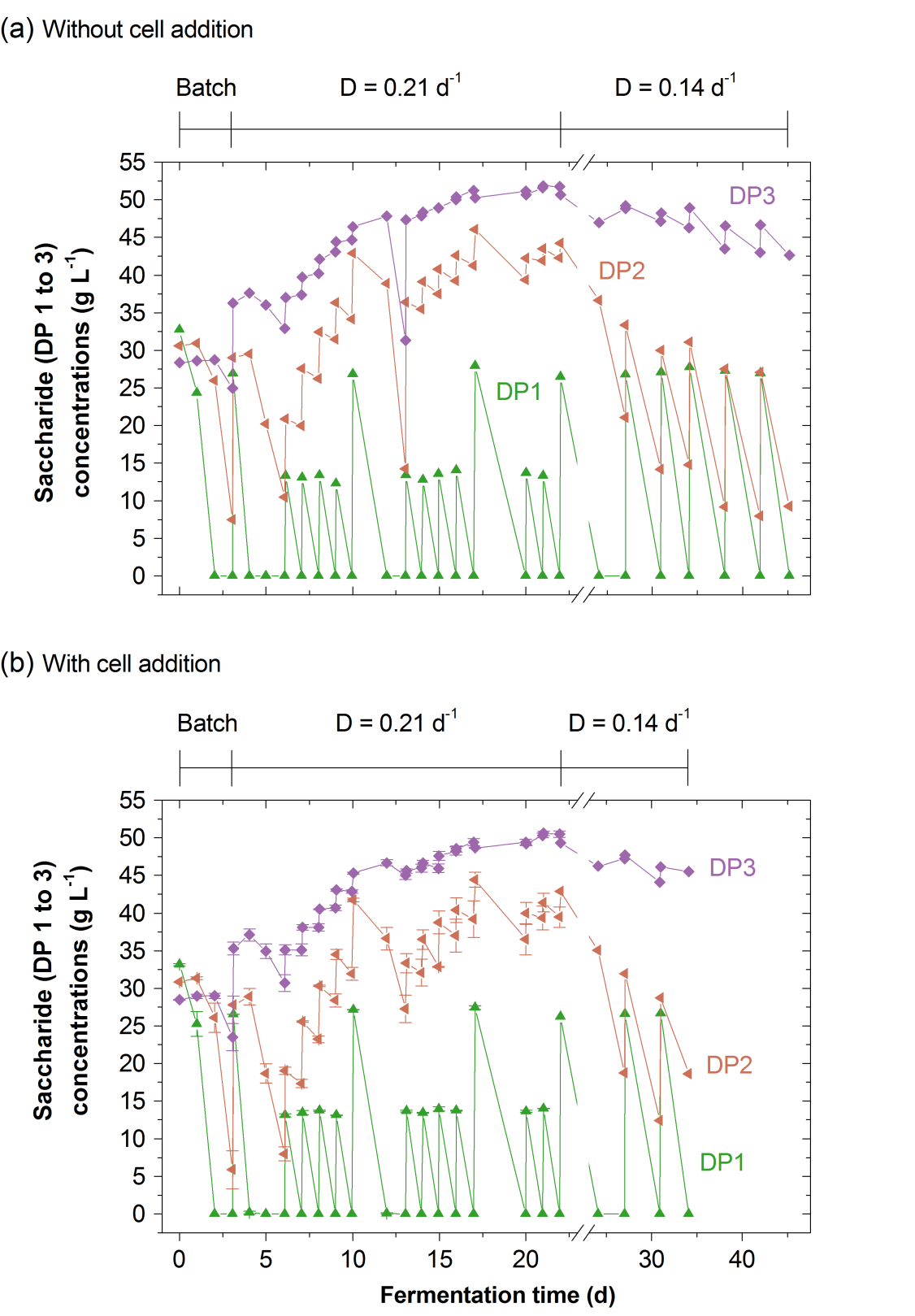


**Figure S1:** Saccharide concentrations in semi-continuous cultivation of *Metschnikowia pulcherrima* on glucose syrup at a high dilution rate (D) on the 2 L scale. (a) Profiles of glucose, maltose and maltotriose concentrations in cultivation of an evolved *M.* *pulcherrima* strain in stirred tank reactors at D = 0.21 d^−1^ on glucose syrup and yeast extract (singlicate), and (b) when additionally preculture was added with every feed (duplicate, mean ± standard error). After 22 days the dilution rate was switched to D = 0.14 d^−1^ (singlicate).

# Figure S2


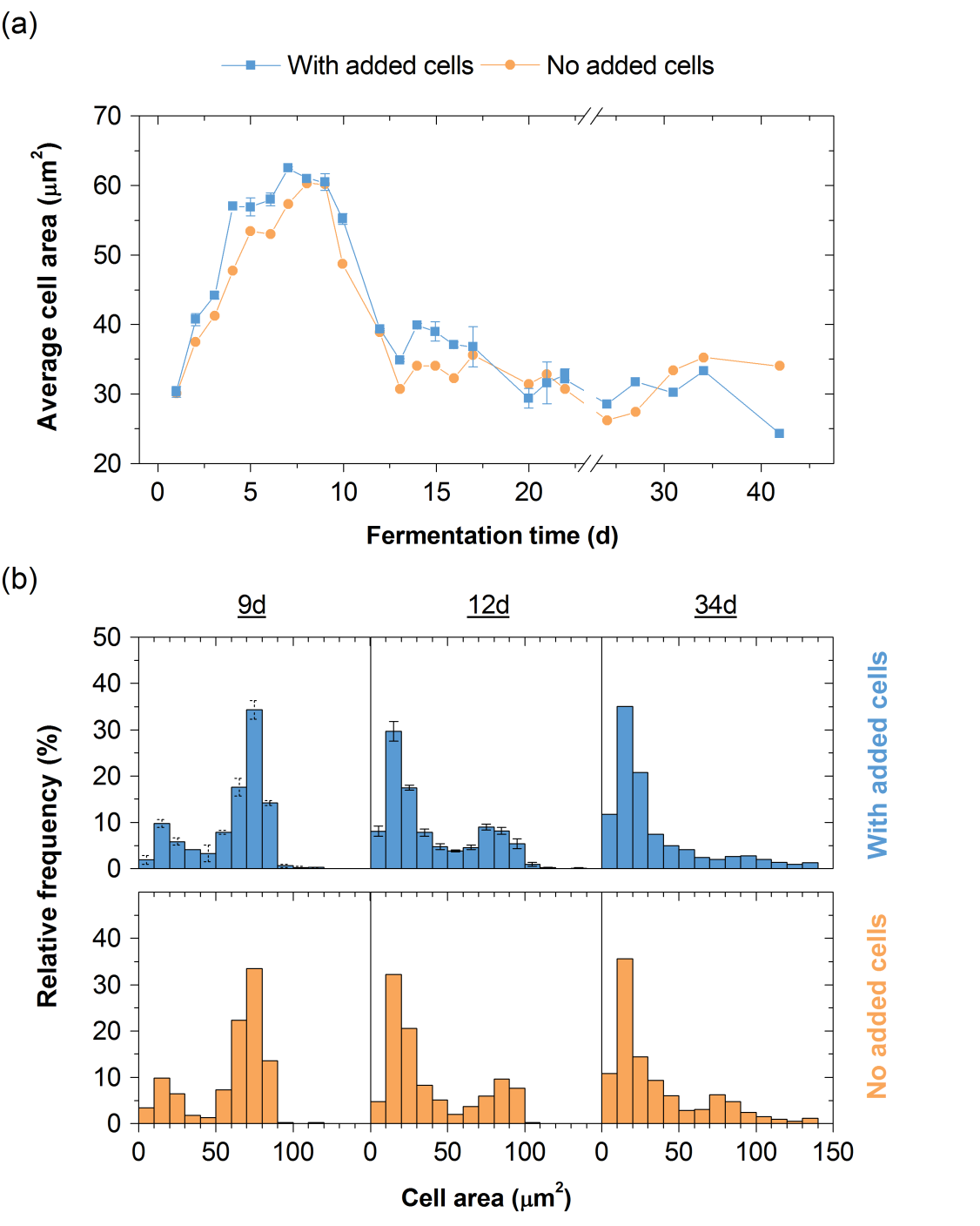


**Figure S2:** Cell size analysis of *Metschnikowia pulcherrima* in semi-continuous cultivation at a high dilution rate (D). (a) Average cell size, and (b) cell size distribution over time semi-continuous cultivations of an evolved *M. pulcherrima* strain in 2 L stirred tank reactors at D = 0.21 d^−1^ on glucose syrup and yeast extract, when additional preculture was either added with every feed (blue; duplicate, mean ± standard error) or not (orange; singlicate).

# Figure S3


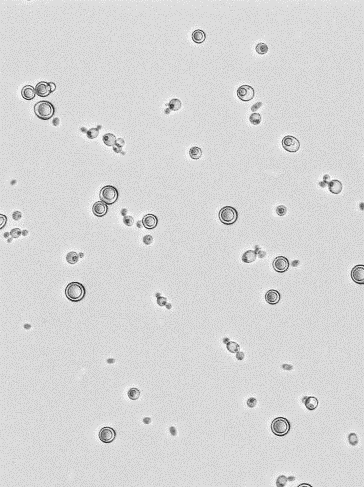

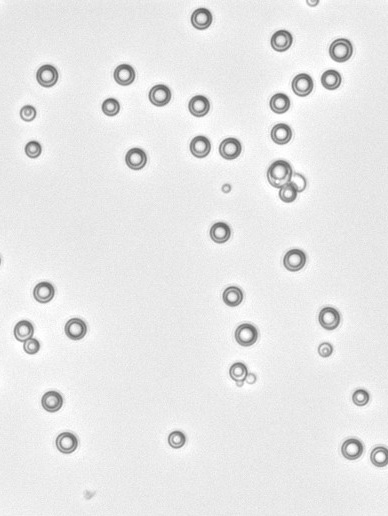

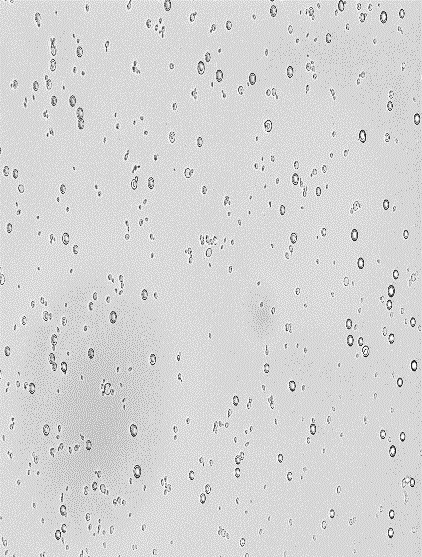
 9 d 12 d 34 d

**
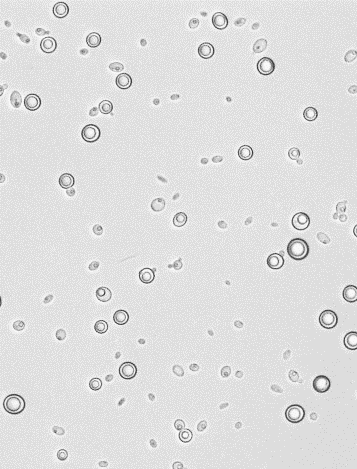

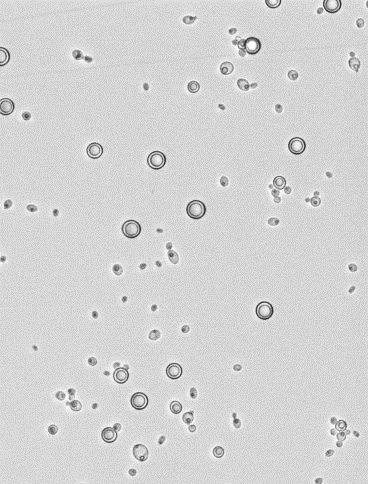

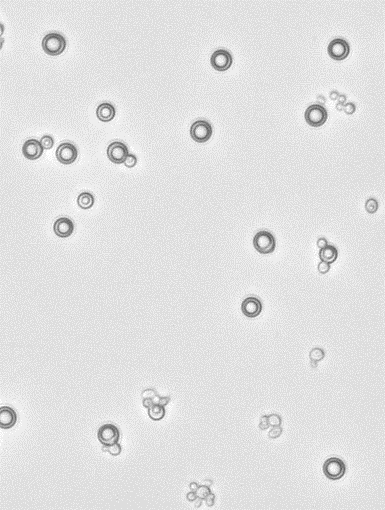
**

**Figure S3:** Micrographs of *Metschnikowia pulcherrima* at different sampling times in semi-continuous cultivation at a high dilution rate (D). The yeast was cultured semi-continuously in 2 L stirred tank reactors at D = 0.21 d^−1^ on glucose syrup and yeast extract. Additional preculture was either added with every feed (blue) or not (orange). The scale bar represents 50 μm.

# Figure S4


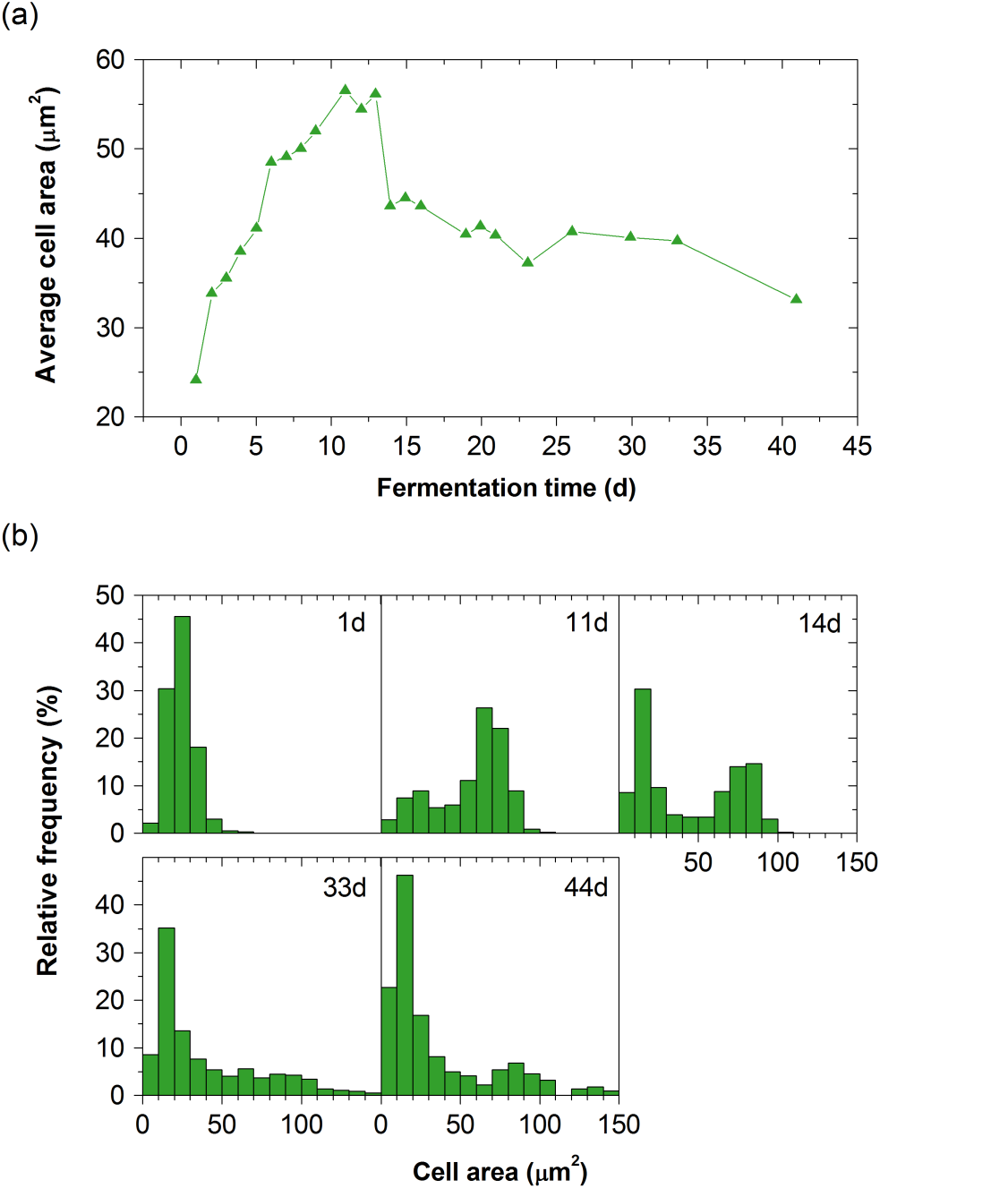


**Figure S4:** Cell size analysis of *Metschnikowia pulcherrima* in semi-continuous cultivation at a lower dilution rate (D). (a) Average cell size, and (b) cell size distribution over time in semi-continuous cultivation of an evolved *M. pulcherrima* strain in 2 L stirred tank reactors at D = 0.14 d^−1^ on glucose syrup and yeast extract (singlicate).

# Figure S5


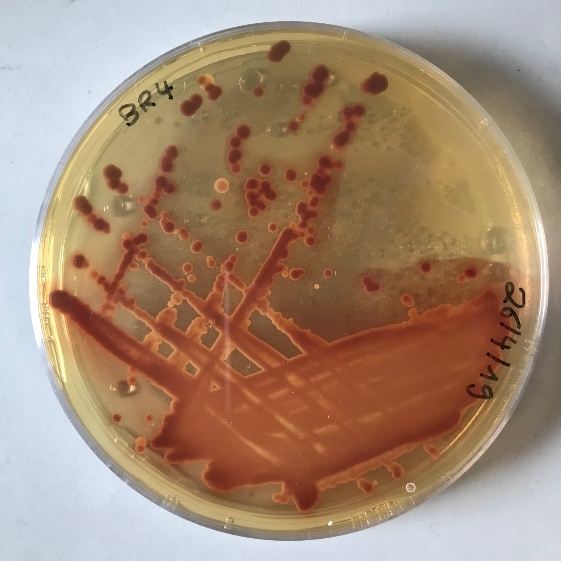


**Figure S5:** *Metschnikowia pulcherrima* plated on an iron-supplemented agar plate. The malt extract agar contains 0.02 mg L^−1^ FeCl_3_. High iron concentrations lead to the increased production of the red pigment pulcherrimin – a distinct characteristic of *M. pulcherrima*.
